# Epigenetic regulation of plant immunity: from chromatin codes to plant disease resistance

**DOI:** 10.1007/s42994-023-00101-z

**Published:** 2023-03-17

**Authors:** Si-Si Xie, Cheng-Guo Duan

**Affiliations:** 1 Shanghai Center for Plant Stress Biology and CAS Center for Excellence in Molecular Plant Sciences, Chinese Academy of Sciences, Shanghai, 200032 China; 2 University of the Chinese Academy of Sciences, Beijing, 100049 China

**Keywords:** Histone modification, DNA methylation, Transcriptional reprogramming, Plant immunity, Disease resistance

## Abstract

Facing a deteriorating natural environment and an increasing serious food crisis, bioengineering-based breeding is increasing in importance. To defend against pathogen infection, plants have evolved multiple defense mechanisms, including pathogen-associated molecular pattern (PAMP)-triggered immunity (PTI) and effector-triggered immunity (ETI). A complex regulatory network acts downstream of these PTI and ETI pathways, including hormone signal transduction and transcriptional reprogramming. In recent years, increasing lines of evidence show that epigenetic factors act, as key regulators involved in the transcriptional reprogramming, to modulate plant immune responses. Here, we summarize current progress on the regulatory mechanism of DNA methylation and histone modifications in plant defense responses. In addition, we also discuss the application of epigenetic mechanism-based resistance strategies in plant disease breeding.

## Introduction

### Histone modifications and DNA methylation

In eukaryotes, the genomic information is packaged as nucleosomes, the basic units of chromatin. Each nucleosome is composed of a core histone octamer (two copies of four core histone proteins H2A, H2B, H3, and H4) and 147 bp DNA. The N-terminal tails of these histones are easily accessed and modified with various covalent modifications, such as methylation, acetylation, ubiquitination, phosphorylation, etc. (Kouzarides 2007), a process called histone post-translational modification (PTM). Among them, histone methylation is a well-characterized PTM. Histone methylation usually occurs at lysine and arginine residues with different methyl numbers, including mono-, di-, and tri-methylation (me1/2/3). Histone lysine methylation is a critical and complex epigenetic marker that dynamically controls the transition between different transcriptional states. Another well-studied histone modification is histone acetylation. It is generally assumed that histone acetylation interferes with the interaction within the nucleosome, thereby leading to a more loose chromatin state for transcriptional activation (Shahbazian and Grunstein 2007). These epigenetic marks are dynamically regulated by different factors, including the enzymes that can catalyze/remove (“writers/erasers”) the modification to/from the histone, and the proteins (“readers”) that recognize and link the modification with other molecules. Epigenetic modifications are generally able to implement transcriptional and/or posttranscriptional regulation of such marked genes. More importantly, growing evidence shows that histone modification homeostasis is essential for the plant immunity regulation.

In addition to the modifications on histone tails, various modifications can also occur on the DNA strand, among which the most prominent one is the methylation of the carbon-5 of cytosine (5-mC). DNA methylation can occur in different sequence contexts, including symmetrical CG, CHG, and asymmetrical CHH (H corresponds to A, T, or C) (Henderson and Jacobsen 2007), and be present at promoters, introns, and transposable elements (TEs). In plants, de novo DNA methylation is established by a specific RNA-directed DNA methylation (RdDM) pathway. In *Arabidopsis*, a canonical RdDM model proposes that single-stranded RNA (ssRNA), produced by RNA POLYMERASE IV (Pol IV), can be recognized by RNA-DEPENDENT RNA POLYMERASE 2 (RDR2) to generate double-stranded RNA (dsRNA), which is processed into 24 nt small interfering RNAs (siRNAs) by DICER-LIKE 3 (DCL3). These siRNAs are then loaded onto an RNA-induced silencing complex (RISC) containing the Argonaute (AGO) protein (AGO4/6/9). The nascent scaffold RNA produced by Pol V recognizes the siRNA–AGO complex through sequence pairing. Subsequently, AGO4 interacts with DOMAINS REARRANGED METHYLASE 2 (DRM2), DEFECTIVE IN RNA-DIRECTED DNA METHYLATION 1 (DRD1), and RNA-DIRECTED DNA METHYLATION 1 (RDM1) to methylate the target DNA (Zhang et al. 2018a). In addition to the de novo establishment of CHH methylation, DNA methylation can also be maintained by different pathways. The symmetric CG methylation is maintained by METHYLTRANSFERASE1 (MET1) and CHG methylation by CHROMOMETHYLASE2 and 3 (CMT2 and CMT3) in *Arabidopsis* (Zhang et al. 2018a). The maintenance of asymmetric CHH methylation requires either CMT2 or RdDM (Huettel et al. 2006; Liu et al. 2014). DECRESED DNA METHYLATION 1 (DDM1), a chromatin remodeling protein, is also required for the maintenance of symmetric methylation (Zemach et al. 2013). DNA methylation is highly correlated with H3K9 methylation and forms a positive feedback loop. In this loop, the H3K9me2-containing nucleosome can be recognized by the BAH domain of DNA methyltransferase CMT2/3 to confer non-CG methylation of the target DNA. In turn, non-CG methylation can be recognized by the SAR domain of SUVH4/5/6 histone methyltransferases to enhance the deposition of H3K9me2 (Duan et al. 2018). In plants, the removal of DNA methylation is mainly catalyzed by a pathway termed active DNA demethylation. In *Arabidopsis*, four demethylases are encoded, including REPRESSOR OF SILENCING1 (ROS1), DEMETER (DME), DEMETER-LIKE 2 (DML2), and DML3. The “chromatin codes” are generally composed of histone modification, DNA modification, histone variants, and ranges of noncoding RNA. In this review, we primarily focus on the mechanism of histone modification and DNA methylation in plant immunity regulation.

### Plant immune pathways

In nature, plants are generally exposed to a complex environment with a range of organisms and microorganisms, including insects, bacteria, fungi, and viruses. All these challenges have important influences on many aspects of plant life, including growth, development, crop yield, and adaptability to the environment. To adapt to these diverse biotic stresses, plants have evolved intricate mechanisms to recognize the characteristics of insects or microorganism and activate the appropriate immune response. Here, cell surface-localized pattern-recognition receptors (PRRs) can recognize the pathogen- or microbe-associated molecular patterns (PAMPs or MAMPs), such as bacteria flagellin or fungal chitin, and induce PAMP-triggered immunity (PTI) (Bigeard et al. 2015). However, pathogens can gradually escape from the host’s monitoring systems, due to long-term coevolution of microorganisms and plants. Therefore, plants evolved resistance (R) proteins to specifically recognize the effectors, delivered from pathogens, which activates another immune response called effector-triggered immunity (ETI) (Jones and Dangl 2006).

The PTI and ETI use different PRRs and intracellular nucleotide-binding domain leucine-rich repeat containing receptors (NLRs), respectively. However, they share some downstream effects, such as the activation of mitogen-activated protein kinase (MAPK) cascades, reactive oxygen species (ROS) generation, hormone signaling transduction, and transcriptional reprogramming. Recent studies demonstrate that the influence of PTI and ETI appears to be mutual and the upregulation of PTI components is also a feature of ETI (Ngou et al. 2021; Yuan et al. 2021). But how ETI can regulate PTI, or how PTI affects ETI still needs to be further explored.

**Fig. 1:**
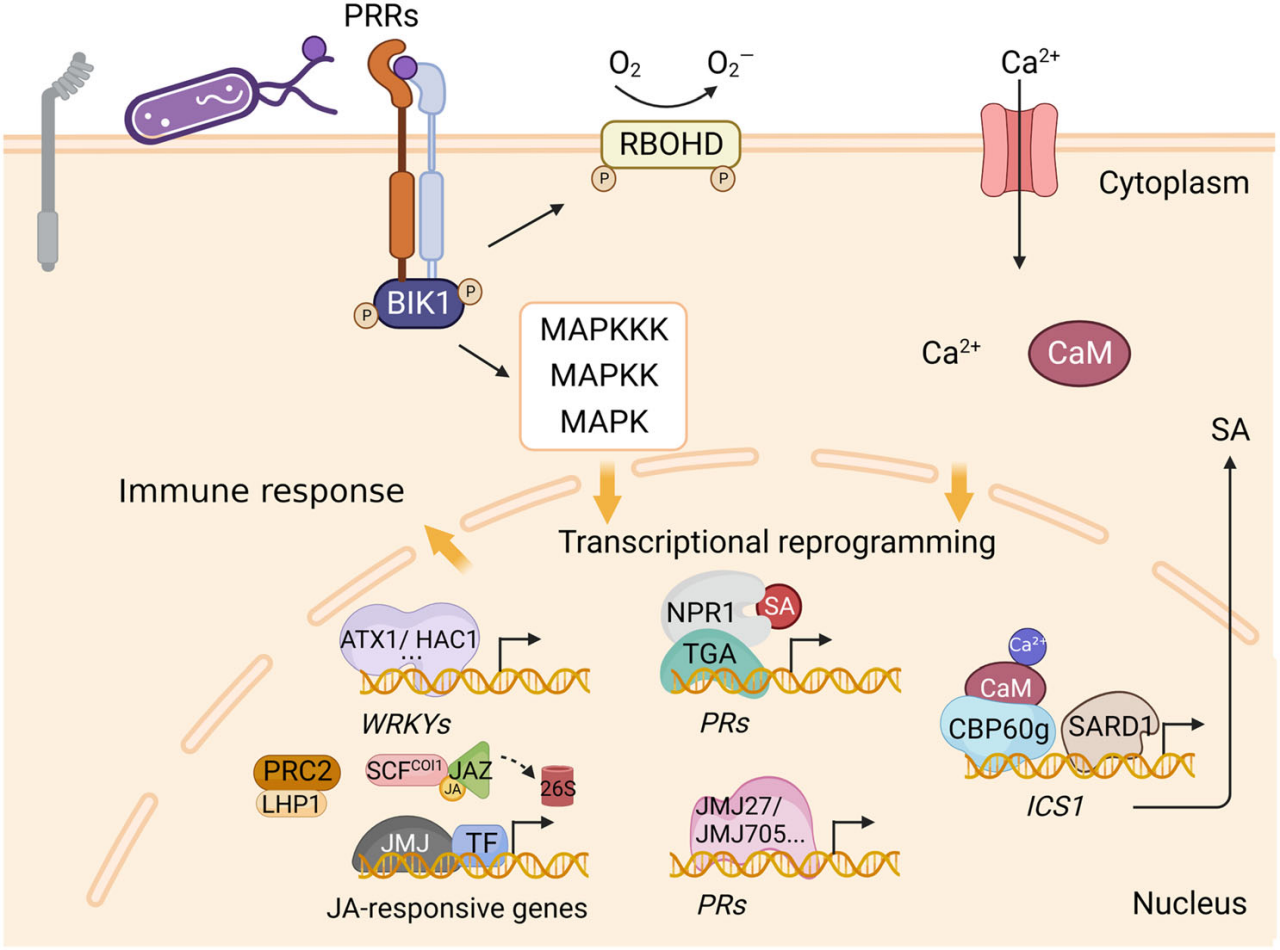
Pathogen-triggered transcriptional reprogramming in the plant immune response. BIK1 is quickly phosphorylated upon PRR recognition of the elicitor, such as flg22, chitin, lectin, etc. Subsequently, several signaling events are activated, such as a Ca^2+^ burst, ROS production, and MAPK cascade, resulting in transcriptional reprogramming in the nucleus. Epigenetic regulators, such as ATX1 and HAC1, are required for activation of the *WRKY*s. The PRC2 complex and JAZ promote silencing of the JA-responsive genes, whereas JMJ functions in their activation. In addition, a Ca^2+^ signal is transduced by Ca^2+^ binding with CaM, followed by binding to other proteins, such as CBP60g, to facilitate expression of the SA biogenesis gene, *ICS1*. The SA receptor, NPR1, recognizes SA and is then translocated into the nucleus to recruit the transcriptional activator, TGA, thereby promoting the expression of *PR* genes. Activation of *PR* genes can also be mediated by histone modifier genes, JMJ27 and JMJ705

Plant hormones are well known as important regulator of plant growth, development and stress responses (Pieterse et al. 2009). In the last two decades, increasing evidence has demonstrated that the classical plant hormones, salicylic acid (SA), jasmonic acid (JA), and ethylene (ET) play key roles in the plant immune response. Generally, SA is considered to participate in the defense against biotrophic pathogens, whereas JA/ET usually function in defense against necrotrophic pathogens. The biosynthesis and perception pathways of these hormones are quite well studied. In the SA signaling pathway, accumulation of SA can result in transformation of the SA receptor, NONEXPRESSOR OF PR GENES 1 (NPR1), from an inactive to active form, followed by its translocation into the nucleus to facilitate expression of the SA-dependent defensive genes, such as *PATHOGENESIS-RELATED GENE 1* (*PR1*), during pathogen infection (Ding and Ding 2020) (Fig. 1). SA is also an important regulator of systemic acquired resistance (SAR), which refers to the phenomenon by which infection of plant aerial tissues, by pathogens, results in the systemic induction of a long-lasting and broad-spectrum disease resistance. Accumulation of SA and activation of the downstream signaling pathway are essential for SAR establishment (Kachroo and Robin 2013).

In the JA signaling pathway, jasmonoyl-L-isoleucine (JAIle), the active form, is repressed by jasmonate ZIM-domain (JAZ) in the resting state. Once JA accumulates, JAIle can recognize CORONATINE INSENSITIVE 1 (COI1) of the SCF^COI1^ complex, leading to the degradation of JAZ by the 26S proteasome. JAIle functions as a transcriptional activator to promote the expression of JA-responsive genes, such as *JASMONATE INSENSITIVE 1* (*JIN1*/*MYC2*) and its downstream genes (Fig. 1). Moreover, release of repression from JAZ leads to the activation of two transcriptional activator factors, ETHYLENE-INSENSITIVE 3 (EIN3) and ETHYLENE-INSENSITIVE3-LIKE 1 (EIL1), thereby promoting expression of another branch of downstream JA-responsive genes, such as *ETHYLENE RESPONSE FACTOR 1* (*ERF1*), *OCTADECANOID-RESPONSIVE ARABIDOPSIS AP2 59* (*ORA59*), and *PLANT DEFENSIN 1.2* (*PDF1.2*) (Li et al. 2022; Ruan et al. 2019).

Under normal conditions, ETHYLENE-INSENSITIVE 1 (ETR1), the receptor for the gaseous hormone ET, activates CONSTITUTIVE TRIPLE RESPONSE 1 (CTR1) to repress the positive regulator, EIN2, via phosphorylation. Upon perception of ET, the release of repression from CTR1 results in the activation of EIN2, which then inhibits the degradation of EIN3 and EIL1, and further activates downstream ET-responsive genes, such as *ERF1* and *ORA59* (Li et al. 2019b).

The antagonism between the SA and JA signaling pathways is well established. In *Arabidopsis*, NPR1 is required for the activation of many transcription factors, such as the TGACG-binding transcription factors (TGAs) and WRKYs, which are responsible for the suppression of JA-responsive genes (Zhang et al. 2018b). In addition, some WRKYs, such as WRKY50, WRKY51, and WRKY70, have also been shown to repress the expression of JA-responsive genes, via NPR1-independent pathways (Gao et al. 2011; Li et al. 2004). Moreover, SA can repress the JA pathway through inhibition of the transcriptional activities of *MYC2* and *ORA59* in *Arabidopsis* (Aerts et al. 2021). In turn, the JA pathway can also exert a repressive effect on the SA pathway. For instance, three transcriptional factors ANAC019, ANAC055, and ANAC072, which function in suppression of the SA biosynthesis enzyme, isochorismate synthase 1 (ICS1), need to be activated by MYC2 (Gimenez-Ibanez et al. 2017). By sharing some common regulators, such as EIN3 and EIL1, the JA and ET pathways are synergistic (Liu and Timko 2021).

## Histone modifications in plant immunity regulation

### Histone methylation in plant immunity regulation

Generally, histone H3 lysine 4 trimethylation (H3K4me3) and H3K36me2/3 are associated with transcriptionally active regions, whereas H3K9me2 and H3K27me3 are associated with silenced regions. H3K4me is catalyzed by a conserved protein complex (COMPASS-like complex) and is mainly located in euchromatin. Seven SET domain proteins (SDGs), including ARABIDOPSIS TRITHORAX 1 (ATX1/SDG27), ATX2 (SDG30), ATX3 (SDG14), ATX4 (SDG16), ATX5 (SDG29), ARABIDOPSIS TRITHORAX-RELATED7 (ATXR7/SDG25), and ATXR3 (SDG2), are proposed to mediate the deposition of H3K4 methylation in *Arabidopsis*.

A number of H3K4 methyltransferases have been implicated in plant immunity regulation. ATX1 has been identified as a ‘master regulator’ in activating expression of the transcription factor, WRKY70, by promoting H3K4me3 deposition (Alvarez-Venegas et al. 2007) (Fig. 1). ATX1 may indirectly activate *PR1* and repress *THI2.1* expression, thereby contributing to a rapid plant response to pathogen infection (Alvarez-Venegas et al. 2007). ATXR7, a Set1 class H3K4me methyltransferase, was reported to be implicated in regulation of PTI, ETI, and SAR immune pathways, together with a H3K36 methyltransferase, SDG8 (Lee et al. 2016). These authors observed that *atxr7* and *sdg8* mutants display enhanced susceptibility to *Botrytis cinerea*, *Pseudomonas syringae pv tomato* DC3000 (*Pst* DC3000), or *Alternaria brassicicola* infection.

Of importance, the *atxr7 sdg8* double mutant showed additive susceptibility, compared with the single mutants, suggesting that ATX7 and SDG8 function synergistically in the regulation of plant immunity. ATXR7 and SDG8 regulate plant immunity partially through controlling the expression of *CAROTENOID AND CHLOROPLAST REGULATION 2* (*CCR2*) and *FACELESS POLLEN 1* (*FLP1/CER3*), two genes that are associated with the biosynthesis of carotenoids and cuticle integrity, respectively. Similar with *sdg8*, *atxr7*, and the *atxr7 sdg8* double mutant, dysfunction of CCR2 and CER3 displays increased susceptibility to *B. cinerea* and *A. brassicicola* (Lee et al. 2016). Several SDG8 studies also reported that SDG8 plays critical roles in plant defense against necrotrophic fungal pathogens and hemi-biotrophic pathogens, via activating JA/ET signaling pathway marker genes, *PDF1.2a*, *VSP2*, *MKK3*, *MKK5*, and the *R* gene, *LAZ5*, respectively (Berr et al. 2010; Palma et al. 2010). Loss of function of *SDG8* results in faster hypersensitive responses (HRs) to *Pst* DC3000 and *Pst* DC3000 *hrpA* strains (De-La-Pena et al. 2012).

In plants, removal of the histone methyl group is achieved through two classes of demethylases, Jumonji C domain-containing proteins (JMJs) and LSD1-like (LDL) proteins (Jiang et al. 2007; Lu et al. 2008). In *Arabidopsis*, the H3K4 demethylase, JMJ14, positively modulates plant immunity and represses gene expression of the negative regulator *SUPPRESSOR OF NPR1-1 INDUCIBLE 1* (*SNI1*), via removing the H3K4me3 from the locus. In addition, JMJ14 was also shown to be required for systematic defense. Loss of function of *JMJ14* leads to attenuation in the local defense response, and reduced Pip accumulation in distal leaves during pathogen invasion (Li et al. 2020).

In *Arabidopsis*, four *LDL* genes (*LDL1-4*) have been identified. Among them, *LDL4/FLOWERING LOCUS D* (FLD)*/REDUCED SYSTEMIC IMMUNITY1* (*RSI1*) is required for the activation of *WRKY29* and *WRKY6* genes, through H3K4me3 dynamics, and differential influences on the expression of *WRKY38*, *WRKY65* and *WRKY53* (Singh et al. 2014b). Furthermore, GLUTATHIONE S-TRANSFERASE THETA 2 (GSTT2), a member of the glutathione S-transferase theta class, was shown to be associated with LDL4 and functions in activating SAR, probably through influencing H3KAc and H3K4me2/3 levels at *WRKY6* and *WRKY29* (Banday and Nandi 2018). Subsequently, it was shown that LDL4 acts as a positive regulator of plant defense against the necrotrophic fungi *B. cinerea* and *Alternaria alternata*. More importantly, the *ldl4* mutants are partially defective in JA signaling, but hyperactive in ethylene signaling (Singh et al. 2019). Recently, the *ldl1 ldl2* double mutant was shown to exhibit resistance to *Pst* DC3000, which may be caused, in part, by H3K4me3-dependent upregulation of *WRKY22/40/70* genes (Noh et al. 2021). In rice, another H3K4me2/3 demethylase, JMJ704, was shown to be a positive regulator in plant defense against *Xanthomonas oryzae pv. Oryzae* (*Xoo*). Here, JMJ704 represses the expression of a subset of negative regulators in plant defense, such as *NRR*, *OsWRKY62*, and *Os-11N3*, by removing H3K4me2/3 and maintaining a transcriptionally inactive state (Hou et al. 2015).

In higher plants, Polycomb group (PcG) proteins associate with different proteins to form multiple protein complexes, named Polycomb Repressive Complex 2 (PRC2) and PRC1, which synergize to maintain gene silencing. The core components of PRC1/2 are conserved in animals and plants. Three H3K27 methyltransferases of the PRC2 complexes have been identified in *Arabidopsis*, including MEDEA (MEA), CURLY LEAF (CLF), and SWINGER (SWN). Recently, MEA was shown to negatively regulate plant immunity. Overexpression of *MEA* results in enhanced susceptibility to *B. cinerea*, *Pst* DC3000 and *Pst-AvrRpt2*. In addition, MEA is associated with a transcription factor, DROUGHT-INDUCED 19 (DIL9), and is recruited to the promoter of *RESISTANT TO P. SYRINGAE 2* (*RPS2*) to repress its expression by deposition of H3K27me3, leading to an attenuated defense response (Roy et al. 2018).

The LIKE HETEROCHROMATIN PROTEIN 1 (LHP1), a subunit of PRC1 responsible for H3K27me3 recognition, acts as a repressor of the MYC2-dependent immune pathway. The *lhp1* mutant displays reduced SA content and is more susceptible to *Pst* DC3000 (Ramirez-Prado et al. 2019). A recent study revealed that the histone modifications H3K27me3 and H3K4me3 work together to affect expression of stress-responsive genes to respond to powdery mildew in hulless barley (Zha et al. 2021). In *Arabidopsis*, REF6, an H3K27me3 demethylase, has been shown to form positive feedback with HEAT SHOCK TRANSCRIPTION FACTOR A2 (HSFA2) to maintain the activation of *HSFA2* and degradation of SUPPRESSOR OF GENE SILENCING 3 (SGS3), during transgenerational inheritance, and the degradation of SGS3 could result in reduced trans-acting siRNA (tasiRNA) production. The REF6-HSFA2 loop and reduced tasiRNA converge to release HEAT-INDUCED TAS1 TARGET 5 (HTT5), which drives early flowering and attenuates immunity (He 2019). In rice, *JMJ705* encodes an H3K27me2/3 demethylase, and JMJ705-mediated H3K27me demethylation is required for basal and induced expression of disease resistance genes (Li et al. 2013). *JMJ705 is* induced during pathogen infection, and *JMJ705* loss of function results in enhanced susceptibility to *Xoo*. Moreover, JMJ705 dynamically removes H3K27me3 from responsive genes, such as *JAMYB*, *PR10*,* TPS3*, and *Os07g11739*, during MeJA induction (Fig. 1).

H3K9me is a typical heterochromatin marker that generally associates with DNA methylation. In *Arabidopsis*, H3K9me2 and H3K9me1 predominantly exist, whereas H3K9me3 is barely detected. KRYPTONITE (KYP)/SU(VAR)3–9 homolog 4 (SUVH4) was the first identified H3K9 methyltransferase and it functions, partially redundantly with SUVH5 and SUVH6, in catalyzing H3K9 methylation in plants (Ebbs and Bender 2006; Jackson et al. 2002; Zhang et al. 2023). A recent study showed that SUVH4/5/6 represses the expression of *PRR/NLR* genes and downstream associated defense genes. The *suvh4 suvh5 suvh6* triple mutant displays greater resistance to *Pst* DC3000 than wild type plants (Cambiagno et al. 2021). In addition, SUVH4 was also shown to be involved in the regulation of pathogen-induced programmed cell death (Dvorak Tomastikova et al. 2021).

**Fig. 2:**
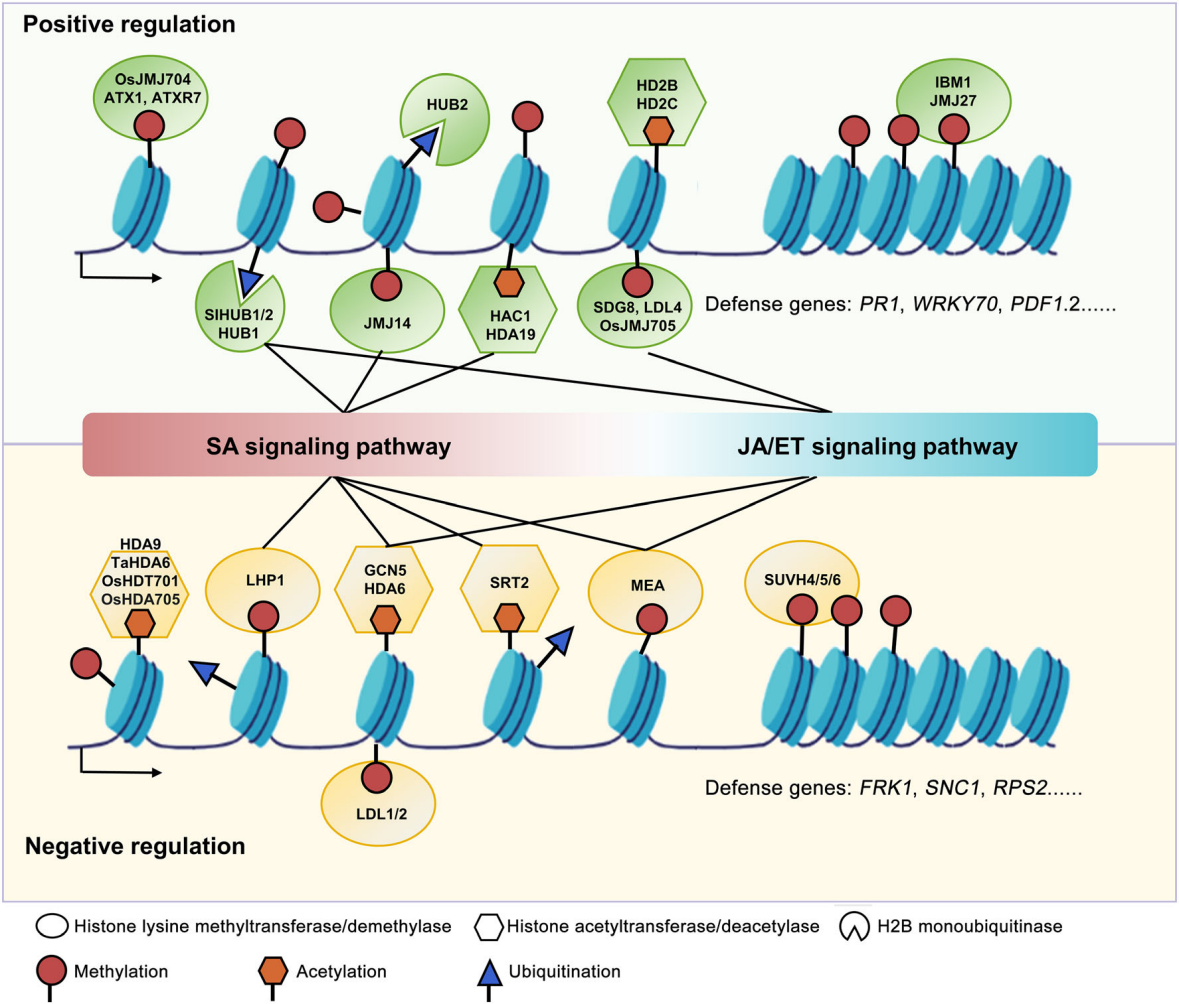
The effects of histone modifiers in plant immune regulation. Histone modifiers are widely implicated in the regulation of an immune response through dynamically modulating the expression of master regulatory genes in hormone (SA, JA/ET) signaling pathways. According to their effects on plant resistance, histone modifiers are divided into two categories: positive and negative regulation. The green icons represent positive regulators, whereas the yellow icons represent negative regulators, in the regulation of disease response

The IBM1, a major H3K9 demethylase in *Arabidopsis*, also participates in plant immunity regulation. The *ibm1* mutants are hyper-susceptible to the bacteria pathogen *Pst* DC3000. IBM1 could directly target defense genes *PR1*, *PR2*, and *FLG22-INDUCED RECEPTOR-LIKE KINASE 1* (*FRK1*) and activate their expression during pathogen infection (Chan and Zimmerli 2019). However, a very recent study showed that the *ibm1* mutant displays increased resistance to *Pst* DC3000 (Lv et al. 2022). The JMJ27, a H3K9 demethylase, is required for resistance to virulent *Pst* DC3000. In this case, JMJ27 functions as a negative mediator of the defense repressor gene, *WRKY25*, and a positive regulator of *PR* genes (Dutta et al. 2017). All above studies show histone methyltransferases and demethylases are widely involved in plant immune regulation (Fig. 2).

### Histone acetylation in plant immunity regulation

Histone acetylation also plays vital roles in immunity regulation (Fig. 2) and is catalyzed by distinct HAT families, including GENERAL CONTROL NONDEREPRESSIBLE 5 (GCN5)-RELATED ACETYLTRA NSFERASE (GNAT), p300/CREB (cAMP-RESPONSIVE ELEMENT-BINDING PROTEIN)-BINDING PROTEIN (CBP), TATA-BINDING PROTEIN-ASSOCIATED FACTOR 1 (TAFII250), and MOZ-YBF2/SAS3-SAS2/ TIP60 (MYST) (Pandey et al. 2002). GCN5, a catalytic subunit of the acetylating modification complex, Spt-ADA-Gcn5-Acetyltransferase (SAGA), was previously shown to influence H3K14ac and H3K9ac level on its targets, but is not strictly coupled to transcriptional activation of the target genes (Benhamed et al. 2008). A recent study proposed that GCN5 has a dual role in transcriptional regulation and repression of SA-mediate immunity. Dysfunction of GCN5 leads to a decrease of H3K14ac in the 5’end of down-regulated targets, and an increase in the 3’ ends of up-regulated targets. This suggests that GCN5 could either activate or repress gene expression by controlling H3K14ac distribution on its target genes. Moreover, GCN5 functions as a repressor of SA-mediated immunity by reducing SA accumulation (Kim et al. 2020). An earlier study showed that a *Phytophthora* effector, PsAvh23, could affect the assembly of the SAGA complex by breaking the association of GCN5 and regulatory subunit Alteration/Deficiency in Activation 2 (ADA2) and suppressing the activation of defense genes in soybean (Kong et al. 2017).

In addition to the GNAT family, a member of the p300/CBP family, HISTONE ACETYLTRANSFERASE OF THE CBP FAMILY 1 (HAC1) and HAC5 also play important roles in immune response. The HAC1/5 can form a complex with NPR1 and TGAs and this HAC–NPR1–TGA complex can then be recruited to *PR* genes to facilitate transcription, through deposition of H3Ac, and a response to SA-triggered immunity (Jin et al. 2018).

Based on their homology to yeast, histone deacetylases (HDACs) can be divided into three groups: REDUCED POTASSIUM DEPENDENCY3 (RPD3), HISTONE DEACETYLASE1 (HDA1), and SIRTUIN2 (Pandey et al. 2002; Yang and Seto 2003). In addition, type-II HDAC (HD2) also has histone deacetylation activity, but is plant-specific (Dangl et al. 2001). HDA19, an RPD3 type histone deacetylase in *Arabidopsis*, can be induced by JA, ET, wounding, and pathogens. Overexpression of *HDA19* causes increased expression of *PR* genes and enhanced resistance to *Alternaria brassicicola* (Zhou et al. 2005). WRKY38 and WRKY62, two members of WRKY group III transcription factors, are required for transcriptional activation and SA-mediated suppression of JA signaling, with functional redundancy (Kalde et al. 2003; Mao et al. 2007). HDA19 can interact with WRKY38 and WRKY62 to repress their transcriptional activation activity. Moreover, the *had19* mutant displays increased susceptibility to *Pst* DC3000 (Kim et al. 2008). However, it has also been reported that *HDA19* loss of function causes increased SA content and increased expression of a group of genes required for accumulation of SA and *PR* genes, such as *PR1* and *PR2*, resulting in enhanced resistance to *Pst* DC3000 (Choi et al. 2012). A similar mechanism was also shown in the regulation of HDA6-mediated plant immunity. HDA6 constitutively represses the expression of pathogen‐responsive genes, including *PR1* and *PR2*, through decreasing histone acetylation levels at their promoters (Wang et al. 2017). The following study showed that this may be caused by HDA6-mediated suppression of SA biosynthesis. HDA6 can directly bind to the promoter regions to repress the expression of *CALMODULIN-BINDING PROTEIN 60 g* (*CBP60g*) and *SYSTEMIC-ACQUIRED RESISTANCE-DEFICIENT 1* (*SARD1*) through histone deacetylation (Wu et al. 2021). A very recent study reported that the acetylation level of TOPLESS is antagonistically regulated by GCN5 and HDA6 to respond to the regulation of JA signaling (An et al. 2022).

TOPLESS is a conserved Groucho/thymidine uptake 1 (Gro/Tup1) family corepressor and is required for the repression of JA-responsive gene expression (Pauwels et al. 2010). GCN5-mediated acetylation of TOPLESS facilitates TPL–NINJA interaction and recruitment to the promoter of MYC2 targets for gene repression. Conversely, HDA6-mediated deacetylation of TOPLESS weakens the TPL–NINJA interaction and activates expression of a JA-responsive gene (An et al. 2022).

In wheat, TaHDA6 interacts with TaHOS15 and is recruited to defense responsive genes, including *TaPR1*, *TaPR2*, *TaPR5*, and *TaWRKY45*, to fine-tune defense responses to powdery mildew (Liu et al. 2019). HDA9 is also a member of the RPD3-like group and interacts with HOS15 to function as a negative regulator of immunity. Importantly, HOS15 can repress these *NLR* genes, including *SUPPRESSOR OF NPR1-1, CONSTITUTIVE1 (SNC1)*, under both pathogen infection and the resting state. However, HDA9 can only repress the *NLR* genes during pathogen infection (Yang et al. 2020). This suggests that HDA9 is involved in the repression of *NLR* genes during a response to pathogen infection, and there may be other factors involved in the repression of *NLR* genes, by HOS15, in the resting state. Interestingly, pathogens have also developed antagonism strategies for better infection. A recent study showed that a secreted fungal effector, UvSec117, can target the rice histone deacetylase OsHDA701 and negatively regulate rice broad-spectrum resistance against rice pathogens (Chen et al. 2022).

SRT2, a NAD^+^-dependent deacetylase of the SIRTUIN2 and HD2 family, is able to negatively regulate plant basal defense against *Pst* DC3000, via suppressing SA biosynthesis. The expression of key master regulators in the SA biosynthesis pathway, including *PAD4*, *EDS5*, and *SID2*, was increased in the *srt2* mutant and decreased in *SRT2* overexpression plants (Wang et al. 2010). In rice, the HD2 subfamily histone deacetylase HDT701 negatively regulates plant innate immunity by modulating histone H4 acetylation of *PRR* and defense-related genes in response to *Xoo* infection (Ding et al. 2012). Additionally, an *Arabidopsis* HD2 class of H3K9ac deacetylase, HD2B, was identified to be targeted by MAP kinase MPK3 and plays an important role in bacteria defense. In this case, MPK3 directly phosphorylates HD2B, thereby conferring its relocation to the nucleus to regulate H3K9 acetylation levels of biotic stress response genes (Latrasse et al. 2017). Furthermore, HD2C functions as a positive regulator in defending against *Cauliflower mosaic virus* (CaMV) infection. Loss of function of *HD2C* results in an increased histone acetylation level on the viral mini-chromosomes, which caused enhanced susceptibility to CaMV. Intriguingly, the P6 protein of CaMV could destroy the function of HD2C through interfering with the HD2C–HDA6 interaction (Li et al. 2021b).

### Histone ubiquitination and other modifications in plant immunity regulation

Compared to histone methylation and acetylation, the function of histone ubiquitination in regulating plant immunity has been less explored. However, the limited studies indicate the important involvement of histone ubiquitination in the plant defense response (Fig. 2). HISTONE MONOUBIQUITINATION1 (HUB1), a RING E3 ligase of histone 2B monoubiquitination, was reported to be an important regulator of plant defense against necrotrophic fungal pathogens (Dhawan et al. 2009). In addition, HUB1 and another E3 ligase, HUB2, have roles in the depolymerization of cortical microtubules, through positively regulating the expression of key protein tyrosine phosphatase genes and promoting protein tyrosine phosphorylation during the defense response to *Verticillium dahliae* toxins (Hu et al. 2014). Additionally, HUB1 and HUB2 can upregulate expression of the *R* gene, *SNC1*, by promoting the deposition of H2B monoubiquitination, and are required for autoimmune responses in the *bon1* mutant (Zou et al. 2014). In tomato, SlHUB1 and SlHUB2 can positively regulate plant defense response to *B. cinerea* through modulating the balance between the SA- and JA/ET-mediated signaling pathways (Zhang et al. 2015).

In rice, an emerging post-translational modification lysine 2-hydroxyisobutyrylation (K_hib_) has been implicated in plant immunity. Histone deacetylases, OsHDA705, OsHDA716, OsSRT1, and OsSRT2, are all responsible for the removal of K_hib_ marks. Among them, OsHDA705 was further shown to negatively regulate rice disease resistance. Dysfunction of OsHDA705 enhanced resistance to *Ustilaginoidea virens*, the bacterial blight pathogen *Xoo* and the rice blast fungus, *M. oryzae*. Importantly, histone Khib functions as an active marker for gene transcription, and is involved in regulating the expression of *R* genes (Chen et al. 2021).

## DNA methylation in plant immunity regulation

**Fig. 3:**
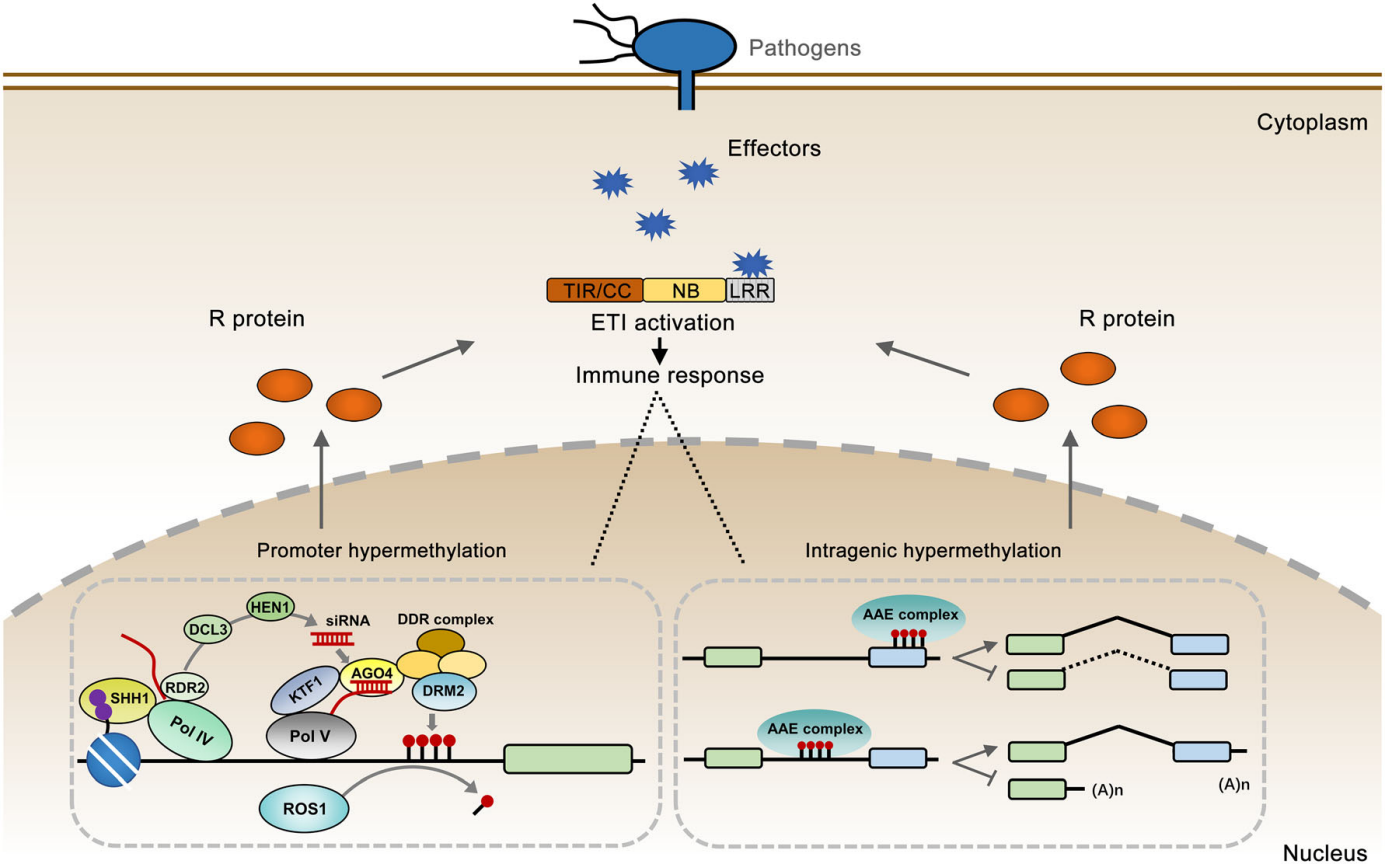
DNA methylation-dependent regulation of *R *gene-mediated immunity. Specific pathogens cause plant disease through the secretion of effectors into host cells. In the resting state, the promoter region of *R* genes is hyper-methylated and silenced by the RdDM pathway. The DNA demethylase, ROS1, can antagonize the silencing of *R* genes through DNA demethylation, during infection, thereby promoting the expression of *R* genes. In another case, the intragenic hyper-methylation can recruit the AAE protein complex to promote the production of full-length transcripts of *R* genes. Impairment of DNA methylation, or the AAE complex, results in mis-splicing or proximal polyadenylation, facilitating the production of intact R protein. Then, the R protein can activate ETI immune responses through recognition of the effectors. The ETI immune response may also have an important effect on the dynamic regulation of the DNA methylation state on *R* genes

The important participation of DNA methylation in plant immunity regulation has been well established (Fig. 3). For example, the methylation level of viral DNA is decreased in *Arabidopsis* 5-mC-deficient mutants after geminivirus infection. Consistently, a number of DNA methylation- and RdDM-deficient mutants have been shown to display enhanced susceptibility to geminiviruses (Raja et al. 2008). Loss of function of NRPD2, the second largest subunit of Pol IV and Pol V, also leads to increased susceptibility to the necrotrophic fungal pathogens *B. cinerea* and *Plectosphaerella cucumerina*. The other mutants involved in different steps of the RdDM pathway, such as *nrpe1*, *ago4*, *drd1*, *rdr2*, and *drm1 drm2*, have similar phenotypes with *nrdp2* during pathogen infection (Lopez et al. 2011). Through DNA methylation sequencing of plants exposed to different biotic stresses, Dowen et al. (2012) reported that the DNA methylation changes in repetitive sequences, or transposons, could affect the expression of neighboring genes in response to SA stress. The CG methylation mutant (*met1-3, ddm1*) and non-CG methylation mutants (*ddc*, *drm1-2 drm2-2 cmt3-11, rdr1*, *rdr2*, *rdr6*, *drd1*, *nrpd1a*, and *dcl2 dcl3 dcl4*) displayed enhanced resistance to *Pst* DC3000.

In recent years, whole-genome bisulfite sequencing studies have revealed that DNA hypomethylation is often associated with enhanced resistance during pathogen infection. For example, in wheat, infection with *Blumeria graminis f. *sp.* tritici* resulted in a significant decrease in CHH methylation and downregulation of *AGO4a* (Geng et al. 2019). Consistent with these findings, in *Arabidopsis*, infestation with the green peach aphid leads to DNA hypomethylation in hundreds of loci, particularly transposable elements (Annacondia et al. 2021). In addition, upon treatment with nematode-associated molecular patterns from different nematode species, or the bacterial pathogen-associated molecular pattern, flg22, both rice and tomato plants displayed global DNA hypomethylation. Intriguingly, hypomethylation mainly occurred in CHH methylation (Atighi et al. 2020). Apart from the factors associated with the RdDM pathway and enzymes that catalyze methylation, *Arabidopsis* ELONGATOR SUBUNIT 2 (ELP2) was shown to be required for pathogen-induced rapid transcriptome reprogramming, through altering methylation levels of specific methyl cytosines (Wang et al. 2013). In addition, MED18, a subunit of mediator, is associated with NRPD2 to regulate the immune response through modulating the expression of defense-related genes (Zhang et al. 2021a).

It would seem that plants undergo a global hypomethylation upon perceiving pathogen signals. For example, flg22 (bacteria elicitor) can trigger the downregulation of a series of RdDM gene expression, including *AGO4*, *AGO6*, *NRPD2*, *NRPD7*, *Nuclear RNA Polymerase E7* (*NRPE7*), *NRPE5*, *INVOLVED IN *DE NOVO* 2* (*IDN2*), *KOW DOMAIN-CONTAINING TRANSCRIPTION FACTOR 1* (*KTF1*), *DRD1*, and *MET1*. The downregulation of these genes results in hypomethylation within the RdDM loci during flg22 induction. Moreover, DNA demethylase ROS1 facilitates the demethylation of an RdDM target (also a disease resistance gene) *TNL RESISTANCE METHYLATED GENE 1* (*RMG1*) and is associated with the activation of a SA-dependent defense response (Yu et al. 2013). Consistent with this finding, the DNA demethylase triple mutant, *rdd* (*ros1 dml2 dml3*), displays increased susceptibility to the fungal pathogen, *Fusarium oxysporum.* In addition, DNA demethylases can positively regulate the expression of stress response genes enriched with transposon or repeat sequence in their promoter regions for defense against fungal pathogens (Le et al. 2014). Intriguingly, among those defense genes mis-expressed by pathogen infection in a *ros1* mutant, only a few were accompanied by DNA methylation changes (Sanchez et al. 2016). Hence, the molecular mechanism of how ROS1 mediates transcriptional reprogramming in immune response has been a mystery. However, recently, Halter et al. (2021) reported that ROS1 can directly bind to the promoters of *RMG1* and *ORPHAN RECEPTOR-LIKE PROTEIN 43* (*RLP43*) and catalyze DNA demethylation during flg22 induction, thereby indirectly promoting the binding of WRKY transcriptional factors to the W-box motif of *RLP43* and activating gene expression.

The gene-for-gene resistance model proposes that one avirulence gene in distinct races of microorganisms can be recognized by genetically interacting with the corresponding *R* gene in plants, thereby leading to plant disease resistance (Dangl and Jones 2001). The largest class of *R* genes encodes a nucleotide-binding site plus leucine-rich repeat (NB-LRR) class of proteins. *R* genes are usually clustered in regions enriched for TEs and repetitive sequences, wherein 5-mC and H3K9me2 are densely deposited. These repressive markers can prevent TE activation to facilitate the integrity of *NB-LRR* genes and stabilize chromatin structure.

DNA hypomethylation may promote the recombination and evolution of *R* genes (Alvarez et al. 2010). Therefore, DNA methylation homeostasis is essential for *R* gene expression and plant resistance (Fig. 3). PigmS, a rice NLR receptor, was reported to suppress the PigmR-mediated broad resistance to pathogen by interfering with the formation of PigmR homodimerization. The *PigmS* promoter contains two tandem miniature transposons, MITE1 and MITE2. The expression of *PigmS* was affected by DNA methylation level in MITE1 and MITE2 mediated by the RdDM pathway. The lower DNA methylation in MITE1 and MITE2 increased the gene expression of *PigmS,* and further compromised PigmR-mediated resistance (Deng et al. 2017).

It is generally accepted that cytosine methylation of the promoter region often plays a repressive role in modulating expression of the gene. However, an earlier study showed that promoter DNA methylation plays a novel enhancing role in resistance to the pathogen. For example, the fungal pathogen, *Magnaporthe grisea*, can induce the expression of *Pib*, an NLR gene in rice. Notably, the DNA methylation level in the promoter region (contains heavy CG methylation) of *Pib* is increased after infection by this fungal pathogen (Li et al. 2011). Furthermore, some studies have shown that DNA methylation not only represses gene expression but also activates gene expression at different targets (Harris et al. 2018; Shibuya et al. 2009). Collectively, these studies indicate that DNA methylation is involved in the regulation of plant immunity, through balancing the transcriptional repression and activation effects to fine-tune the expression of different defensive genes.

DNA methylation not only regulates the expression of *R* genes, but also modulates the length of the *R* gene transcript (Fig. 3). *RPP7*, which encodes a CC-NB-LRR protein and contains a *Ty-1* COPIA-type retrotransposon (also named *COPIA-R7*), is specifically enriched with the H3K9me2 marker in the sense orientation of the first intron, contributes to disease resistance during *Hyaloperonospora parasitica* infection (Eulgem et al. 2007). ENHANCED DOWNY MILDEW 2 (EDM2), Anti-silencing 1 (ASI1) and ASI1 immunoprecipitated protein 1 (AIPP1) form a protein complex (designated as AAE complex) that recognizes and affects the H3K9me2 at *COPIA-R7* to promote the 3’ distal polyadenylation (Duan et al. 2017; Lei et al. 2014; Tsuchiya and Eulgem 2013). The intronic heterochromatin has been shown to be required for the distal polyadenylation of the *RPP7* gene. Abnormal expression of the *RPP7* transcript occurred in the *ddm1* and *suvh4 suvh5 suvh6* mutants (Zhang et al. 2021b). Similarly, another *R* gene, *RPP4*, was also shown to be regulated by the AAE complex (Deremetz et al. 2019; Zhang et al. 2021b). The *RPP4* partially overlaps with a *COPIA4‐like* retrotransposon (*AT4TE42860*), at its 3′ terminal exon region, and facilitates disease resistance to *Hyaloperonospora parasitica* (Garcia et al. 2010). Dysfunction of this AAE complex leads to the mis-splicing of the *RPP4* transcript (Zhang et al. 2021b). These examples support the notion that intragenic heterochromatin, caused by TE insertions, serve as an important regulatory element in *R* gene expression.

## Epigenetic regulation in defense priming and transgenerational SAR

Plants can respond faster or show more resistance to environment challenges when previously exposed to a moderate stress. After suffering from mild environment stress, plants will be primed and form a ‘memory’ to be better equipped to cope with the situation when again encountering this stimulus. Priming involves changes in histone modifications, DNA methylation and accumulation of inactive MAPKs and transcriptional factors (Beckers et al. 2009; Jaskiewicz et al. 2011; Luna and Ton 2012; Singh et al. 2014a). For example, H3 and H4 acetylation, as well as H3K4 methylation, on the *WRKY* promoter may promote priming of genes during pathogen infection or treatment with the SA synthetic analog, acibenzolar S-methyl (Jaskiewicz et al. 2011). Interestingly, abiotic stress could trigger the priming of biotic stress. For example, the *hac1-1* mutant displays increased susceptibility to *Pst* DC3000 after repetitive stress (cold, salt, and heat), but there was no obvious distinction, compared to wide type, during nonstress. Moreover, the expression of PTI-responsive genes *WRKY53*, *FRK1*, and *NHL10* failed to be activated in the *hac1* mutant, after repetitive heat stress (Singh et al. 2014a). It has been reported that CHROMATIN ASSEMBLY FACTOR 1 (CAF1), a histone chaperone, mediates the repression of priming of defensive genes, under non-inductive conditions. Consistent with this model, dysfunction of CAF1 resulted in spurious activation of SA-dependent defense response accompanied with low nucleosome occupancy and high H3K4me3 at the transcription start sites of defensive genes (Mozgova et al. 2015).

Some studies have provided evidence showing that DNA methylation is implicated with transgenerational SAR. An earlier finding showed that the next generation of tobacco displays enhanced resistance to TMV, *Pseudomonas syringae*, or *Phytophthora nicotianae,* after infection during the first generation. TMV infection can enhance homologous recombination frequency and induce expression of *PR1* and promote callose accumulation to mediate resistance against the pathogen in the second generation (Kathiria et al. 2010). A subsequent study showed that descendants could acquire resistance after the infection with *Pst* DC3000, in the first generation. This transgenerational SAR effect is regulated by the RdDM pathway and transmitted by hypomethylation at CHG sites (Luna and Ton 2012). These findings suggest that the inheritance of a specific DNA methylation pattern may contribute to the regulation of transgenerational SAR. However, how DNA methylation contributes to transgenerational SAR remains to be elucidated. It is possibly that the DNA methylation state and the accumulated of effector proteins, in the first generation, will be gamete-transmitted to the next generation, or re-established in the second generation.

## Application of epigenetics to plant immunity

**Fig. 4:**
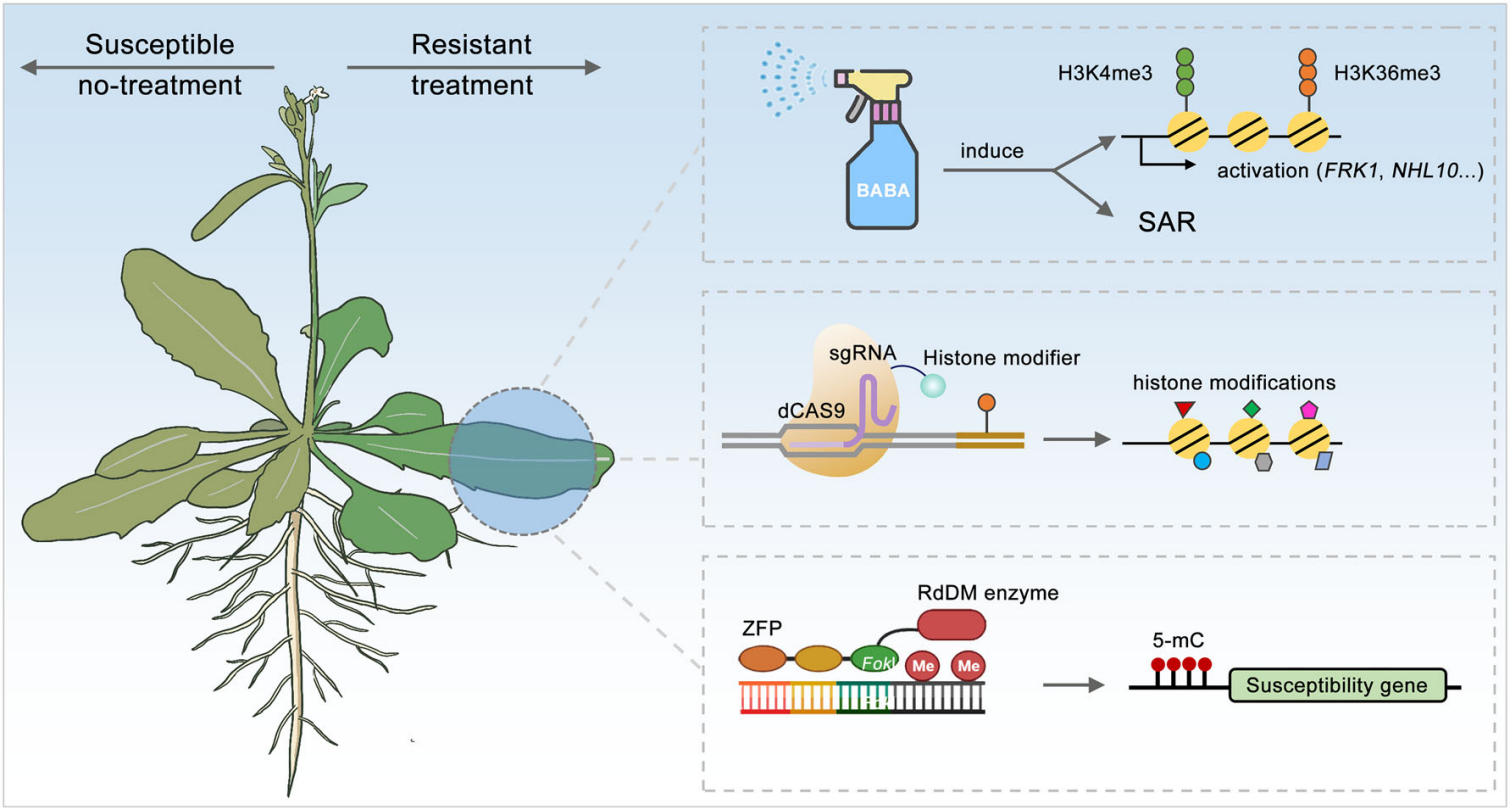
Epigenetic regulation-based plant disease resistance strategy. Spraying a chemical reagent, BABA, onto pants can induce high expression of defensive genes (such as *FRK1*, *NHL10*) through facilitating the deposition of H3K4me3 and H3K36me3. In addition to affecting the expression of immune-related genes, BABA can also induce SAR to enhance plant resistance during a pathogen infection. dCAS9 could be used as a tool for activating or repressing the histone modifiers and further fine-tune plant immune response, via modulating expression of downstream defensive genes. Zinc-finger-fused epigenetic regulators can also be utilized for promoting the establishment of DNA hyper-methylation, through the RdDM pathway, in the promoter regions to repress expression of the susceptibility genes and improve the tolerance to pathogen infection in future. ZFP, zinc finger protein

To survive in adverse conditions, plants have evolved diverse mechanisms to enhance resistance to pathogens. Epigenetic regulation gradually becomes an efficient tool to overcome the challenges from biotic stress. Epigenetic mechanism-mediated crop disease resistance strategies are being employed in crop breeding (Fig. 4). For instance, some chemical agents have been identified that enhance plant resistance through an epigenetic mechanism. In rice, application of 5-azadeoxycytidine, a DNA de-methylating agent, enhances plant resistance to the bacterial pathogen *Xanthomonas* (Akimoto et al. 2007). Additionally, in plants, the chemical SAR inducer β-aminobutyric acid (BABA) was shown to enhance resistance to various pathogens, including the hemi-biotrophic bacterium *Pst* DC3000, the necrotrophic fungus *B. cinerea*, and the oomycete pathogen *P. parasitica*, through affecting the histone modification and inducing defensive gene expression (Martinez-Aguilat et. 2016; Zimmerli et al. 2000, 2001). Moreover, BABA induces resistance against *P. parasitica* in transgenic NahG (salicylate hydroxylase) plants. Thus, BABA mainly activates the SAR pathway downstream of SA accumulation (Zimmerli et al. 2000). Another study showed that BABA treatment induces the deposition of callose, stomatal closure, and expression of the defensive gene, *PR1*, in the SA pathway and other PTI-responsive genes, including those that facilitate resistance to the necrotrophic bacterium, *Pectobacterium carotovorum *subsp.* carotovorum* (*Pcc*), in *Arabidopsis* (Po-Wen et al. 2013).

In common bean, BABA treatment can prime the expression of many defense genes. Interestingly, the levels of several histone modifications, such as H3K4me3 or H3K36me3, are higher in the promoter regions for different defense genes, during 24 h after BABA treatment, but are not accompanied by obvious accumulation of defensive gene transcripts. The actual activation of defensive genes occurs 24 h after BABA treatment (Martinez-Aguilat et. 2016). In potato, histone lysine methylation, but not acetylation, appears to function in BABA-triggered resistance. BABA induces higher level of H3K4me2 in *NPR1*, the positive regulator of SAR, to respond to *Phytophthora infestans* during initial treatment. After a 48 h treatment, *NPR1* was repressed by the accumulation of H3K27me3, whereas *SNI1*, the negative regulator of SAR, was activated by an increase in H3K4me2 level (Meller et al. 2018). BABA has been widely used to improve the disease resistance in crops, such as common bean, potato, grapes, tomato, pepper, cabbage, and fruits (Hamiduzzaman et al. 2005; Janotik et al. 2022; Kim et al. 2013; Li et al. 2019a, 2021a; Martinez-Aguilat et. 2016; Meller et al. 2018). These studies also have shown that enhanced resistance, induced by chemical agents, depends on the regulation of epigenetic markers.

Importantly, DNA methylation and histone modification can be manipulated by several approaches. The SUVH2/9 proteins are two inactive histone methyltransferase, due to their lack of a post SET domain that is required for cofactor and peptide substrate-binding. Johnson et al (2014) used Zinc Finger (ZF) fused with SUVH2/9 to establish DNA methylation, at an unmethylated site, through the RdDM pathway. The *fwa-4*, an unmethylated epiallele of *FWA*, was observed to be successfully methylated and the *ZF–SUVH2/fwa-4* plant displayed an early flowering phenotype. Moreover, the altered DNA methylation could be stably transmitted to the next generation (Johnson et al. 2014). A synthetic ZF, fused to the *Arabidopsis* DEFECTIVE IN MERISTEM SILENCING 3 (DMS3), a component of the RdDM pathway, was employed to deposit DNA methylation at the promoter of a susceptibility (*S*) gene, *MeSWEET10a*, in cassava. Methylation in this promoter prevented the binding of the transcription activator-like (TAL) effector, TAL20, which prevented transcriptional activation of *MeSWEET10a* and showed a decrease in bacterial blight symptoms in these cassava plants (Veley et al. 2023). A similar approach for selectively targeting epigenetic silencing could be developed in future studies to modified key defensive genes for improvement of disease resistance in other crop species (Fig. 4).

In addition, CRISPR/dCas9-mediated manipulation of histone modifications has also been reported in plants. Interfering (CRISPRi) or activation (CRISPRa) of targeted genes is mediated by genetically fusing effector proteins to dCas9. CRISPR/dCas9-mediated histone modification regulation has been applied in response to abiotic stress. For example, HAC1-fused dCas9 transgenic plants were generated to improve drought stress tolerance through the transcriptional activation of the positive regulator, ABSCISIC ACID-RESPONSIVE ELEMENT-BINDING FACTOR 2 (ABF2/AREB1) (Roca Paixao et al. 2019). Therefore, similar strategies could also be employed to engineer plant disease resistance through targeting of key regulators of plant defense. For example, spraying artificial 24-nt siRNAs may be used to induce RdDM pathway-mediated silencing of defense repressive genes to confer immune activation (Fig. 4). This approach deserves further exploration in future.

## Conclusion and perspective

The world today is facing a severe food crisis and a deteriorating natural environment, which highlights the importance of biological breeding in establishing global food security. During evolution, plants utilized both genetic and epigenetic variations to cope with diverse environmental stresses. The growing evidence shows that epigenetic markers could influence and modulate plant disease resistance, and thus epigenetic regulation emerges as an efficient strategy for plant disease resistance breeding. In this review, we summarize the transcriptional reprogramming of defensive gene, mediated by “writers” and “erasers” of histone modifications and DNA methylation, in plant defense responses, and the increasing application of epigenetic mechanisms in improvement of crop yield potential, as well as techniques that have potential for future uses in crop breeding programs.

Over the years, these studies have greatly broadened our understanding of how epigenetic markers mediate plants defense response against pathogen. Both effective plant defense and successful infection by pathogens depend upon the dynamic regulation of epigenetic markers, to activate or repress, the expression of defensive genes. In general, the transcriptional reprogramming, mediated by chromatin modifications, acts downstream of immune signaling pathways. Although many chromatin regulators have been identified to play a role in plant immunity, the reported mechanisms mostly focus on how the specific regulator affects some key defensive genes, through changing the chromatin modifications. However, how these epigenetic factors are recruited by immune factors to defense genes remains largely unsolved. Hence, more detailed regulatory mechanisms need to be further explored in future. Some important questions include: what are the dynamics of epigenetic factors before and after pathogen infection? how do epigenetic regulators induce the plant response at the single cell level after infection? how do plants achieve their return to a resting state after a period of defense? and how do plants transmit/maintain the “stress memory” to the next generation? Answers to these questions will help us gain a deeper understanding of how plants respond to pathogen infection and make corresponding changes at the chromatin level. Such knowledge will facilitate the development of more efficient disease resistance strategies based on epigenetic mechanisms.

## Data Availability

Data sharing not applicable to this article as no datasets were generated or analyzed during the current study.
